# Effect of NPC1L1 and HMGCR Genetic Variants With Premature Triple-Vessel Coronary Disease

**DOI:** 10.3389/fcvm.2021.704501

**Published:** 2021-12-01

**Authors:** Xueyan Zhao, Jingjing Xu, Xiaofang Tang, Keyong Huang, Jiawen Li, Ru Liu, Lin Jiang, Yin Zhang, Dong Wang, Kai Sun, Bo Xu, Wei Zhao, Rutai Hui, Runlin Gao, Lei Song, Jinqing Yuan

**Affiliations:** State Key Laboratory of Cardiovascular Disease, National Center for Cardiovascular Diseases, Fuwai Hospital, Chinese Academy of Medical Sciences and Peking Union Medical College, Beijing, China

**Keywords:** NPC1L1, HMGCR, genetic variants, SYNTAX score, premature triple-vessel disease

## Abstract

**Background:** Both Niemann-Pick C1-like 1 (NPC1L1) and 3-hydroxy-3-methylglutaryl-coenzyme A reductase (HMGCR) play a key role on dyslipidaemia. We aim to evaluate whether NPC1L1 and HMGCR genetic variants are associated with susceptibility of premature triple-vessel disease (PTVD).

**Methods:** Four single-nucleotide polymorphisms (SNPs) (rs11763759, rs4720470, rs2072183, and rs2073547) of NPC1L1; and three SNPs (rs12916, rs2303151, and rs4629571) of HMGCR were genotyped in 872 PTVD patients (males ≤ 50 years old and females ≤ 60 years old), and 401 healthy controls.

**Results:** After adjusting for age and sex, rs12916 of HMGCR was associated with the risk of PTVD in dominance model [odds ratio (OR) = 1.68, 95% confidence intervals (CI): 1.29–2.18, *P* < 0.001], recessive model (OR = 1.43, 95% CI: 1.08–1.90, *P* = 0.013) and codominant model (OR = 1.38, 95% CI: 1.17–1.63, *P* < 0.001); meanwhile, rs4720470 of NPC1L1 was related to increased risk of PTVD in recessive model (OR = 1.74, 95% CI: 1.14–2.74, *P* = 0.013). Patients who carried both variant rs4720470 and rs12916 also had the risk of PTVD (*P* < 0.001); however, there were no correlation between these SNPs and the SNYTAX score (all *P* > 0.05).

**Conclusions:** This is the first report that rs4720470 is a novel polymorphism of the NPC1L1 gene associated with PTVD, and rs12916 of HMGCR gene appears to be a strong genetic marker of PTVD. Our study may improve the early warning, therapeutic strategies and drug development of PTVD.

## Introduction

Patients with triple-vessel disease (TVD) often have diffuse coronary stenosis and a higher risk of death and cardiovascular events (1). Since premature triple-vessel disease (PTVD) has great threat to human health in the world, early diagnosis and timely aggressive therapeutic intervention are particularly important. Present studies show that the increase of the level of low-density lipoprotein cholesterol (LDL-C) is an important pathogenic factor in the development of coronary atherosclerosis (2, 3). The maintenance of intracellular cholesterol homeostasis mainly depended on two regulatory mechanisms: endogenous cholesterol synthesis and exogenous cholesterol uptake by extracellular LDL receptor, the former is catalyzed by 3-hydroxy-3-methylglutaryl-coenzyme A reductase (HMGCR); and Niemann-Pick C1-like-1 (NPC1L1) participates in the absorption of exogenous food cholesterol (4).

At present, it is believed that genetic determinants of LDL-C levels may impose additional risk of atherosclerotic cardiovascular disease (5) and PTVD has a strong genetic correlation (6). In recent years, studies on familial hypercholesterolemia have focused on low-density lipoprotein receptor, apolipoprotein B and proprotein convertase subtilisin/kexin type 9 (PCSK9). However, there are relatively few studies on gene polymorphism of NPC1L1 and HMGCR in patient with PTVD. In addition, it is worth noting that, although genome-wide association studies (GWAS) technology has developed rapidly in recent years, only 2.5% of patients with severe high level of LDL-C carried a known genetic variation in familial hypercholesterolemia (7).

In recent years, the gene polymorphism of NPC1L1 and HMGCR has attracted more and more attention in coronary heart disease (CHD) (8–10). We recently reported for the first time that the rs4720470 on NPC1L1 gene and rs2303151 on HMGCR gene are related to the risk of TVD with 1-year major adverse cardiac and cerebrovascular events (11). However, whether the relevant single nucleotide polymorphisms (SNP) of NPC1L1 and HMGCR genetic variants are associated with susceptibility of PTVD and stenosis severity has never been reported before. Therefore, we performed a study to evaluate the correlation between the SNPs of NPC1L1 and HMGCR with susceptibility of PTVD and the Synergy between Percutaneous Coronary Intervention with Taxus and Cardiac Surgery (SYNTAX) score in Chinese population.

## Materials and Methods

### Patients and Study Variables

**Figure 1:**
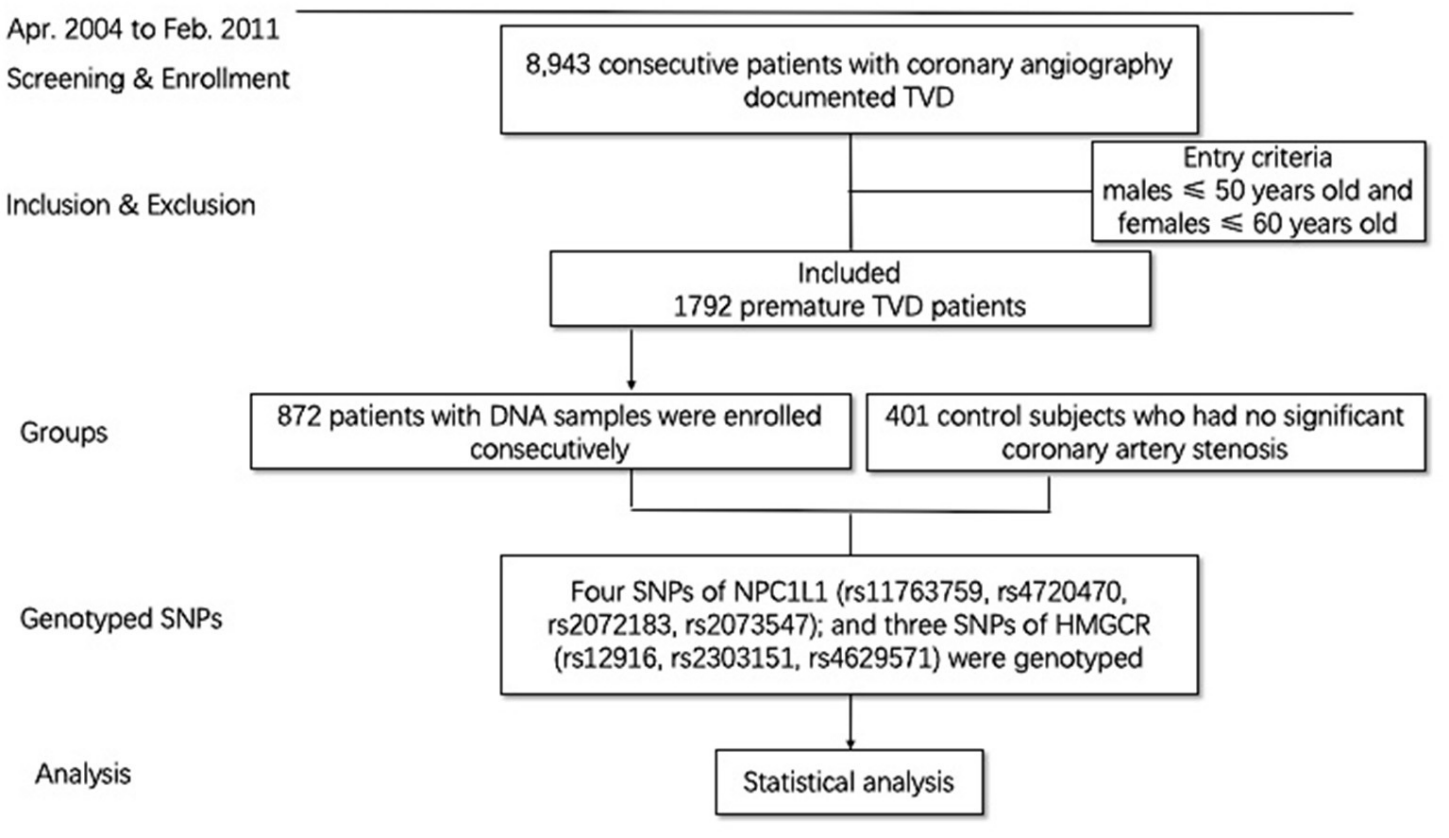
Patient flow chart.

All the cases used in the current study were from a prospective cohort of coronary artery patients. The cohort study consecutively enrolled 8,943 consecutive patients with TVD who were willing to have follow-up in Fuwai Hospital (Beijing, China) from April 2004 to February 2011. Previous study has already described the methodology (12, 13). All the patients underwent angiography during the admission, and all outcome data were systematically collected following angiography. The definition of TVD was angiographic stenosis of ≥50% in all three main coronary arteries, including the left anterior descending, circumflex, and right coronary arteries, with or without the left main artery involved. PTVD is defined as patients with TVD, requiring male ≤ 50 years old and female ≤ 60 years old. A total of 1,792 patients (20.0%) met the PTVD criteria. Among them, 872 patients who had blood samples and met the detection criteria were included in the PTVD group. Six hundred fourteen (70.41%) patients with PTVD were previously treated with statins in the study. All of them were previously prescribed with no ezetimibe because of the time of enrollment (April 2004 to February 2011). The healthy control group was those who had no significant coronary artery stenosis (<50% coronary artery stenosis) by coronary angiography or coronary computed tomography (Figure 1). Baseline data were recorded in a dedicated database by independent research personnel. The SYNTAX score (14) was calculated by experienced cardiologists from an independent angiographic core laboratory (Interventional Cardiovascular Imaging Core Laboratory, National Center for Cardiovascular Diseases, Beijing, China). The study abided by the Declaration of Helsinki. The ethics committee of Fuwai Hospital approved the research protocol. Written informed consent was obtained from all participants.

### Blood Sampling and Determination of SNPs

Blood samples were drawn from fasting patients within 24 h after admission, genomic DNA was extracted from peripheral blood leukocytes by the standard salting-out method (15). The samples were then stored in a refrigerator at −80°C until testing. After consulting the previous relevant literature and HapMap project (http://hapmap.ncbi.nlm.nih.gov/) of the Chinese with a minor allele frequency ≥0.05, the following SNPs were selected, including 4 NPC1L1 sites: rs11763759, rs4720470, rs2072183, and rs2073547 and 3 HMGCR sites: rs12916, rs2303151, and rs4629571. The SNPs were genotyped using an improved multiplex ligation detection reaction technique (16).

### Statistical Analysis

The baseline characteristics of the study participants were presented as means with corresponding standard deviations for continuous variables or as percentages for categorical variables. To compare the baseline characteristics between PTVD cases and controls, two-sample *t*-test was used for continuous variables, Pearson Chi-square test for categorical variables. Univariate or multivariate adjusted logistic regression model adjusting age and sex was used to estimate the odds ratio (OR) and 95% confidence intervals (CI) for risk of PTVD events associated with selected SNPs. According to the case-control design, we assumed the odds ratio of 1.5, with probability of 0.5 in the control group, statistical power of 0.8 at two-sided significance level of 0.05, the required sample size is 388 for cases and 388 for controls. Thus, the sample size in our study meets the current research objectives. All the statistical analyses were performed using SPSS software version 19.0 (IBM Corporation, Armonk, New York, USA). Tests were two sided with statistical significance set at *P* < 0.05.

## Results

### Baseline Characteristics

A total of 1,273 persons were finally included (Figure 1). Among them, there were 872 patients with PTVD (mean age, 47.71 ± 6.12 years), and 401 healthy control subjects (mean age, 46.91 ± 10.16 years). Compare with the control group, patients with PTVD were more male, higher BMI, higher rate of smoking history, lower heart rate, lower systolic and diastolic blood pressure, higher triglycerides, lower high-density lipoprotein cholesterol and LDL-C, higher glucose, creatinine and hypersensitive C-reactive protein (all *P* < 0.05; Table 1).

**Table 1:** Comparison of clinical characteristics between PTVD and controls.

**Variables**	**PTVD (*n* = 872)**	**Control (*n* = 401)**	***P*-value**
Sex, M/F	635/237	267/134	0.027
Age, y	47.71, 6.12	46.91, 10.16	0.087
BMI, kg/m^2^	26.49, 3.36	25.24, 3.44	<0.001
Smoker, %	55.16	33.3	<0.001
HR, bpm	70.76, 10.66	74.13, 12.23	<0.001
SBP, mm Hg	124.44, 18.12	127.18, 20.80	0.018
DBP, mm Hg	78.75, 12.09	80.23, 12.86	0.048
TC, mmol/L	4.82, 1.18	4.71, 0.98	0.068
TG, mmol/L	2.04, 1.16	1.73, 1.21	<0.001
HDL-C, mmol/L	1.00, 0.26	1.21, 0.32	<0.001
LDL-C, mmol/L	2.75, 1.19	2.94, 0.90	0.012
Glucose, mmol/L	6.35, 3.26	5.71, 1.11	<0.001
Creatinine, μmol/L	77.33, 17.11	73.24, 15.33	<0.001
Hs-CRP, mg/L	4.22, 18.31	1.54, 1.87	0.040

*Data are expressed as mean ± standard deviation or percentage (%). PTVD, premature triple-vessel disease; BMI, body mass index; HR, heart rate; SBP, systolic blood pressure; DBP, diastolic blood pressure; TC, total cholesterol; TG, triglycerides; HDL-C, high density lipoprotein cholesterol; LDL-C, low density lipoprotein cholesterol; Hs-CRP, hypersensitive C-reactive protein*.

### The Frequency of Genetic Polymorphism

The frequency of gene polymorphism of 4 SNPs of NPC1L1 (rs11763759, rs4720470, rs2072183, and rs2073547) and 3 SNPs of HMGCR (rs12916, rs2303151, and rs4629571) were compared between PTVD and control group. Chi-square test was used to analyze the difference of genotype frequency between PTVD patients and controls, and the genotype frequencies of rs4720470 (*P* = 0.04) and rs12916 (*P* < 0.001) were significantly higher in the PTVD group than in the control group (Table 2).

**Table 2:** Frequency of genetic polymorphism in case-control patients.

**Gene**	**Polymorphism**	**Genotype**	**PTVD**	**Control**	*P*-value*
NPC1L1	rs11763759	TT	775 (88.98%)	356 (88.78%)	0.98
		CT	91 (10.45%)	43 (10.72%)	
		CC	5 (0.57%)	2 (0.50%)	
	rs4720470	CC	415 (47.65%)	202 (50.37%)	0.04
		CT	356 (40.87%)	171 (42.64%)	
		TT	100 (11.48%)	28 (6.98%)	
	rs2072183	GG	342 (39.31%)	141 (35.16%)	0.35
		CG	407 (46.78%)	198 (49.38%)	
		CC	121 (13.91%)	62 (15.46%)	
	rs2073547	AA	340 (38.99%)	140 (34.91%)	0.35
		GA	408 (46.79%)	197 (49.13%)	
		GG	124 (14.22%)	64 (15.96%)	
HMGCR	rs12916	TT	212 (24.40%)	138 (34.59%)	<0.001
		TC	406 (46.72%)	170 (42.61%)	
		CC	251 (28.88%)	91 (22.81%)	
	rs2303151	CC	558 (63.99%)	262 (65.34%)	0.21
		CT	277 (31.77%)	130 (32.42%)	
		TT	37 (4.24%)	9 (2.24%)	
	rs4629571	AA	719 (82.45%)	330 (82.29%)	0.91
		AG	142 (16.28%)	67 (16.71%)	
		GG	11 (1.26%)	4 (1.00%)	

* *Pearson chi-square test. PTVD, premature triple-vessel disease*.

### Gene Polymorphisms and PTVD Susceptibility

#### SNPs of NPC1L1

For both the PTVD group and control group, the genotype distributions of polymorphisms were conformed to Hardy–Weinberg equilibrium (both *P* > 0.05). In the light of the univariate logistic regression analysis, under the recessive genetic mode, there was a significant correlation between rs4720470 and PTVD, and the risk in PTVD group was 1.73 times higher than that in the control group (OR: 1.73,95% CI: 1.13–2.72, P = 0.014). While there was no statistical difference among rs11763759, rs4720470, or rs2073547 in the three genetic modes between PTVD group and control group (all *P* > 0.05; Table 3).

**Table 3:** Univariate and multivariate adjusted logistic regression analysis of NPC1L1 gene SNPs and the risk of PTVD.

**Polymorphism**	**Model**	**Crude**			Multivariate*		
		**OR**	**95% CI**	***P***	**OR**	**95% CI**	***P***
rs11763759 T>C	Codominant (TT/TC/CC)	0.99	0.70–1.41	0.951	0.995	0.70–1.43	0.976
	Recessive (CC/TT+TC)	1.15	0.25–8.07	0.866	1.20	0.25–8.43	0.832
	Dominance (TT/CT+CC)	0.98	0.68–1.44	0.916	0.98	0.68–1.45	0.934
rs4720470 C>T	Codominant (CC/CT/TT)	1.18	0.99–1.42	0.071	1.19	0.99–1.43	0.062
	Recessive (TT/CT+CC)	1.73	1.13–2.72	0.014	1.74	1.14–2.74	0.013
	Dominance (CC/CT+TT)	1.12	0.88–1.41	0.366	1.12	0.89–1.43	0.330
rs2072183 G>C	Codominant (GG/GC/ CC)	0.89	0.75–1.05	0.168	0.89	0.75–1.06	0.181
	Recessive (CC/GG+GC)	0.88	0.64–1.24	0.464	0.88	0.63–1.23	0.450
	Dominance (GG/CC+GC)	0.84	0.65–1.07	0.157	0.84	0.66–1.08	0.180
rs2073547 A>G	Codominant (AA/AG/GG)	0.88	0.75–1.05	0.161	0.89	0.74–1.05	0.168
	Recessive (GG/AA+AG)	0.87	0.63–1.22	0.417	0.86	0.62–1.21	0.390
	Dominance (AA/AG+GG)	0.84	0.66–1.07	0.163	0.85	0.66–1.08	0.190

* *Adjusted for age and sex. NPC1L1, Niemann-Pick C1-like 1; PTVD, premature triple-vessel disease; OR, odds ratio; CI, confidence interval*.

We further added age and sex to the above-mentioned SNPs for multivariate logistic regression analysis, the results showed that rs4720470 was still an independent risk factor for PTVD in recessive genetic mode (OR: 1.74,95% CI: 1.14–2.74, *P* = 0.013). However, there was no significant difference among other SNPs in the three genetic models (all *P* > 0.05; Table 3).

#### SNPs of HMGCR

For both the PTVD group and control group, the genotype distributions of polymorphisms were conformed to Hardy–Weinberg equilibrium (both *P* > 0.05). According to the univariate logistic regression analysis, the results showed that under each of the three genetic models (codominant, dominant and recessive), there was a significant correlation between rs12916 and PTVD. In the codominant mode, the risk of PTVD was 1.35 times higher than that in the control group (OR: 1.35, 95% CI: 1.15–1.59, *P* < 0.001); in the recessive genetic mode, the risk of PTVD was 1.37 times higher than that in the control group (OR: 1.37, 95% CI: 1.05–1.82, *P* = 0.024), and in the dominant genetic model, the risk of PTVD was 1.64 times higher than that in the control group (OR: 1.64, 95% CI: 1.27–2.12, *P* < 0.001). However, there was no statistical difference among rs2303151 or rs4629571 in the three genetic modes between PTVD group and control group (all *P* > 0.05; Table 4).

**Table 4:** Univariate and multivariate adjusted logistic regression analysis of HMGCR gene SNPs and the risk of PTVD.

**Polymorphism**	**Model**	**Crude**			Multivariate*		
		**OR**	**95% CI**	***P***	**OR**	**95% CI**	***P***
rs12916 T>C	Codominant (TT/TC/CC)	1.35	1.15–1.59	<0.001	1.38	1.17–1.63	<0.001
	Recessive (CC/TC+TT)	1.37	1.05–1.82	0.024	1.43	1.08–1.90	0.013
	Dominance (TT/TC+CC)	1.64	1.27–2.12	<0.001	1.68	1.29–2.18	<0.001
rs2303151 C>T	Codominant (CC/CT/TT)	1.12	0.90–1.39	0.320	1.12	0.91–1.40	0.297
	Recessive (TT/CT+TT)	1.93	0.96–4.30	0.081	1.95	0.97–4.36	0.077
	Dominance (CC/CT+TT)	1.06	0.83–1.36	0.641	1.07	0.83–1.37	0.610
rs4629571 A>G	Codominant (AA/AG/GG)	1.12	0.90–1.39	0.320	1.00	0.76–1.34	0.980
	Recessive (GG/AG+GG)	1.27	0.43–4.60	0.686	1.17	0.39–4.26	0.79
	Dominance (GG/AA+AG)	0.99	0.73–1.35	0.945	0.99	0.73–1.36	0.967

* *Adjusted for age and sex. HMGCR, 3-hydroxy-3-methylglutaryl-coenzyme A reductase; PTVD, premature triple-vessel disease; OR, odds ratio; CI, confidence interval*.

We further added age and sex to the above-mentioned SNPs for multivariate logistic regression analysis, results showed that rs12916 was still an independent risk factor for getting PTVD in codominant model (OR: 1.38, 95% CI: 1.17–1.63, *P* < 0.001), recessive genetic model (OR: 1.43, 95% CI: 1.08–1.90, *P* = 0.013) and dominant model (OR: 1.68, 95% CI: 1.29–2.18, *P* < 0.001). There was no significant difference among other SNP in the three genetic models (all *P* > 0.05; Table 4).

### Combined SNPs of NPC1L1 and HMGCR Analysis

We further analyzed the PTVD risk of patients with both significant SNPs variants at the same time. According to logistic regression analysis adjusted by age and sex, the patients with both variant rs4720470 of NPC1L1 in recessive model and variant rs12916 of HMGCR in codominant model had 1.46 times higher risk of PTVD than the patients with no variants (OR = 1.46, 95% CI: 1.23–1.74, *P* < 0.001); patients with both variant rs4720470 in recessive model and variant rs12916 in recessive model related to the 1.35 times higher risk of PTVD (OR = 1.35, 95% CI: 1.14–1.61, *P* < 0.001); when the patient has both variant rs4720470 in recessive model and variant rs 12916 in dominance model, the risk of PTVD was 1.27 times higher (OR = 1.27, 95% CI: 1.12–1.45, *P* < 0.001; Table 5).

**Table 5:** Univariate and multivariate adjusted logistic regression analysis of combined SNPs of NPC1L1 and HMGCR and the risk of PTVD.

**Combined model**		**Crude**		Multivariate*		
	**OR**	**95% CI**	***P***	**OR**	**95% CI**	***P***
rs4720470 Recessive + rs12916 Codominant	1.44	1.22–1.72	<0.001	1.46	1.23–1.74	<0.001
rs4720470 Recessive + rs12916 Recessive	1.33	1.12–1.58	0.001	1.35	1.14–1.61	<0.001
rs4720470 Recessive + rs12916 Dominance	1.26	1.11–1.43	<0.001	1.27	1.12–1.45	<0.001

* *Adjusted for age and sex. SNP, single nucleotide polymorphisms; NPC1L1, Niemann-Pick C1-like 1; HMGCR, 3-hydroxy-3-methylglutaryl-coenzyme; PTVD, premature triple-vessel disease; OR, odds ratio; CI, confidence interval*.

### Associations of Gene Polymorphisms With SYNTAX Score

SYNTAX score was divided into three groups as low-risk (≤22), medium-risk (23–32), and high-risk (≥33), and further evaluated. Univariate analysis showed that the four SNPs of NPC1L1 and three SNPs of HMGCR were not significantly associated with the SYNTAX score (all *P* > 0.05). After conducted logistic regression analysis adjusted for age and sex, there was no change in the results (all *P* > 0.05; Table 6).

**Table 6:** Univariate and multivariate adjusted logistic regression analysis of NPC1L1 and HMGCR gene SNPs and the SYNTAX score.

**Polymorphism**	**Model**	**Crude**			Multivariate*		
		**OR**	**95% CI**	***P***	**OR**	**95% CI**	***P***
rs11763759 T>C	Codominant (TT/TC/CC)	0.95	0.65–1.37	0.770	0.95	0.65–1.37	0.773
	Recessive (CC/TT+TC)	0.48	0.08–2.88	0.421	0.51	0.08–3.06	0.460
	Dominance (TT/CT+CC)	0.98	0.66–1.46	0.914	0.98	0.66–1.45	0.904
rs4720470 C>T	Codominant (CC/CT/TT)	1.07	0.89–1.29	0.464	1.07	0.89–1.28	0.490
	Recessive (TT/CT+CC)	1.14	0.77–1.68	0.516	1.13	0.77–1.68	0.529
	Dominance (CC/CT+TT)	1.08	0.84–1.39	0.561	1.07	0.83–1.38	0.592
rs2072183 G>C	Codominant (GG/GC/ CC)	1.08	0.90–1.30	0.415	1.08	0.90–1.30	0.423
	Recessive (CC/GG+GC)	1.06	0.74–1.52	0.751	1.06	0.74–1.53	0.745
	Dominance (GG/CC+GC)	1.13	0.87–1.46	0.356	1.13	0.87–1.46	0.370
rs2073547 A>G	Codominant (AA/AG/GG)	1.10	0.91–1.32	0.324	1.09	0.91–1.31	0.334
	Recessive (GG/AA+AG)	1.08	0.76–1.55	0.673	1.08	0.76–1.55	0.660
	Dominance (AA/AG+GG)	1.15	0.89–1.49	0.275	1.15	0.89–1.49	0.294
rs12916 T>C	Codominant (TT/TC/CC)	0.98	0.83–1.17	0.844	0.97	0.82–1.15	0.730
	Recessive (CC/TC+TT)	0.79	0.60–1.05	0.101	0.78	0.59–1.03	0.074
	Dominance (TT/TC+CC)	1.25	0.93–1.68	0.142	1.23	0.92–1.66	0.169
rs2303151 C>T	Codominant (CC/CT/TT)	0.98	0.78–1.22	0.827	0.97	0.78–1.21	0.771
	Recessive (TT/CT+TT)	1.32	0.72–2.43	0.374	1.29	0.70–2.38	0.414
	Dominance (CC/CT+TT)	0.91	0.70–1.19	0.494	0.91	0.70–1.18	0.462
rs4629571 A>G	Codominant (AA/AG/GG)	0.92	0.69–1.25	0.605	0.91	0.68–1.23	0.557
	Recessive (GG/AG+GG)	0.74	0.24–2.33	0.609	0.70	0.22–2.20	0.539
	Dominance (GG/AA+AG)	0.93	0.67–1.30	0.676	0.93	0.67–1.29	0.644

* *Adjusted for age and sex. NPC1L1, Niemann-Pick C1-like 1; HMGCR, 3-hydroxy-3-methylglutaryl-coenzyme A reductase; PTVD, premature triple-vessel disease; OR, odds ratio; CI, confidence interval; SYNTAX, Synergy between Percutaneous Coronary Intervention with Taxus and Cardiac Surgery*.

## Discussion

We analyzed the four SNPs of NPC1L1 and four SNPs of HMGCR in Chinese patients with PTVD and healthy control subjects. The results showed that: (1) To the best of our knowledge, this is the first report that rs4720470 (C>T) of NPC1L1 gene in the recessive genetic model (TT/CT + CC) increased the risk of PTVD. (2) rs12916 (T>C) of HMGCR gene is closely associated with the increased risk of getting PTVD in codominant, recessive and dominant modes. (3) However, there is no correlation between NPC1L1 and HMGCR genetic variants and SYNTAX score in this study.

Exogenous cholesterol can lead to an increase in plasma cholesterol levels through food intake and intestinal absorption. NPC1L1 is an essential protein in intestinal cholesterol absorption and highly expressed in human small intestine and liver (4). Meanwhile, NPC1L1 in liver can promote the retention of cholesterol in bile by hepatocytes and prevent the loss of cholesterol from bile (17). NPC1L1 gene polymorphism can affect the efficiency of cholesterol absorption, and then influence plasma LDL-C concentration. NPC1L1 is also therapeutic target of the lipid-lowering drug ezetimibe (18). Our study showed that the rs4720470 of NPC1L1 was associated with the risk of premature coronary heart disease and TVD, which was 1.74 times higher than that of the control group. Rs4720470 is located on human chromosome 7 and belongs to intron variation. We have not found any previous literature on rs4720470 in PTVD. Previous reports on other SNPs of NPC1L1 and CHD are as follows. Stitziel et al. (19) reported that the inactivation mutation of NPC1L1 could lead to a decrease in the level of plasm LDL-C and a reduction in the risk of CHD. Cohen et al. (20) reported that multiple rare variants of NPC1L1 were correlated with decreased cholesterol absorption and low level of plasma LDL-C. The above studies have shown that the inactivation variation of NPC1L1 can lower the level of LDL-C and reduce the risk of CHD. However, other studies have shown that mutations of NPC1L1 can increase the risk of CHD events. Muendlein et al. (21) reported that rs55837134 of NPC1L1 is associated with increased risk of cardiovascular events in CHD (HR = 1.67). Polisencki et al. (10) investigated the SNPs of NPC1L1 in elderly patients (mean age of 75 ± 3 years) with CHD, data indicated that variation in the NPC1L1 gene is associated with plasma total and LDL-C levels and the risk of CHD events (HR: 1.50–1.67; *P* < 0.02). The rs4720470 of NPC1L1 gene in our study is related to the risk of PTVD. The specific mechanism is not clear. We speculated that the variation of SNP may result in the activation of NPC1L1 gene function, which leads to an increase in the level of LDL-C, and leads to the progression of atherosclerosis, causing premature coronary heart disease. In addition, previous study (22) has shown that individuals with genetic variants could attenuate lipid-lowing response to statins, which was associated with genetic alternative splicing and modulation of microRNAs expression (23, 24), which can also lead to an increased risk of progression of coronary atherosclerosis. Hence, we speculated that the above reasons lead to PTVD. Further functional research should be carried out in the future. In addition, whether different races affect the results is also worthy of further study.

HMGCR is a crucial rate-limiting enzyme of cholesterol synthesis. The increase of HMGCR activity leads to the increase of cholesterol synthesis in the liver. Our study showed that the rs12916 (T > C) of HMGCR gene is correlated with the risk of PTVD in all of codominant, recessive and dominant modes. Especially in the dominant mode, the risk of PTVD was the highest, which was 1.68 times higher than that of the control group (OR: 1.68, *P* < 0.001). This suggests that there is a strong correlation between rs12916 and the risk of PTVD. The rs12916 of HMGCR gene is located on chromosome five and belongs to three prime UTR variants. Previous studies on rs12916 and coronary heart disease are as follows. A study recently published by Kettunen et al. (25) showed that reducing the expression of rs12916 in participants of European ancestries could reduce the risk of CHD, which was consistent with our results in Chinese population. Statin is an inhibitor of HMGCR and a cornerstone drug in the treatment of CHD. A study by Swerdlow et al. (26) also found that rs12916 was related to weight gain associated with statins and an increased risk of type two diabetes. The above studies show the importance of rs12916 in coronary heart disease, which is not only associated with the risk of coronary heart disease, but also associated with adverse side effects of statins. Our results further show that the variation of rs12916 in Chinese population can lead to the risk of early and severe CHD. Our study indicate the importance of rs12916 SNP in coronary heart disease, which is not only related to the risk of coronary heart disease (25) and adverse side effects of statin therapy (26), but more importantly, the variation of this site can lead to the risk of early and severe coronary artery disease. Whether we can develop related treatment for this SNP in the future to reduce the risk of early and severe coronary heart disease is worthy of further study.

Monogenic familial hypercholesterolemia often causes an increase in LDL-C and increases the risk of coronary heart disease (27). However, some patients with hypercholesterolemia have polygenetic causes. Our study showed that a small number patients with PTVD carried both variant of HMGCR and NPC1L1. These patients had an increased risk of PTVD, but the risk seems to be similar to that of a single gene variant. Previous studies on polygenic variation of cholesterol have focused on LDL receptor, apolipoprotein B and PCSK9 genes. Recently, Trinder et al. (5) showed that monogenic (LDL receptor, apolipoprotein B and PCSK9) and polygenic hypercholesterolemia were associated with an increased risk of atherosclerotic cardiovascular disease events compared with the general population. Research by Sharifi et al. (28) showed that, in patients with a diagnosis of familial hypercholesterolemia, those with a monogenic cause had a higher severity of carotid and coronary preclinical atherosclerosis than those with a polygenic etiology. For the first time in our study, we explored the effects of monogenetic variation and combined variation of SNPs in HMGCR and NPC1L1 on the risk of PTVD, and all these variations were associated with genetic susceptibility to PTVD.

SYNTAX score is a clinical evaluation method and often used in patients with multi-vessel coronary artery disease, which reflects the severity and complexity of coronary artery disease and can guide further revascularization strategies (29, 30). However, previous studies have never explored the relationship between HMGCR and NPC1L1 gene variation and SNYTAX score. In this study, we conducted a preliminary work in PTVD population that might be help as a further exploration, and the results showed that there was no correlation between these SNPs and SNYTAX score. The reason can be explained as follows. SYNTAX score is often used to reflects the degree of coronary stenosis. However, the factors such as stenosis site, degree of calcification, bifurcation and complete occlusion are also in the computation of SYNTAX score. In our manuscript, the results showed that there was no correlation between these SNPs and SNYTAX score, which may be related to the above computation of SYNTAX score; and may also be related to the population we selected, because PTVD patients are younger and the SYNTAX score will also progress with the increase of age. In the future, we can expand the sample size to further evaluate the correlation between these SNPs and SYNTAX score.

Exploring the genetic variation of coronary heart disease is of important guiding significance for the mechanism study, screening of high-risk patients of premature CHD as well as the improvement of treatment strategy. For example, drugs targeting PCSK9 show good effects in reducing LDL-C and the risk events of CHD (31, 32). Our study showed that rs4720470 variation of NPC1L1 and rs12916 variation of HMGCR are related to the risk of PTVD, which lead to the increase of genetic susceptibility of PTVD and may be involved in the pathogenesis of PTVD. Its future clinical value lies in the early detection of PTVD patients through these SNPs; and because HMGCR and NPC1L1 are the target sites of statins and ezetimibe in patients with CHD, we can further investigate whether these SNPs can provide clues to the precise treatment of drugs in patients with PTVD. Of course, since many genes are race-specific, larger samples and multi-ethnic studies are needed to further evaluate and verify the results of this study.

## Limitations

Some limitations of our analysis should be considered. First of all, this is a single-center research, which may limit the generalizability of its findings; second, the sample size is relatively small even though a large population is screened; third, the population in this study is selected as the Chinese population, and the results of related genes of different races are deserving of further verification in the future;fourth, we have found the correlation between these SNPs and PTVD, the biological mechanism of related gene polymorphisms has not been studied in this study, and further research is needed in the future.

## Conclusion

Polymorphisms of the HMGCR gene and NPC1L1 gene are associated with the risk of PTVD in Chinese population. For the first time, we report that the rs4720470 of NPC1L1 gene is related with PTVD, and the rs12916 of HMGCR gene is closely related to the risk of PTVD. It is likely to bring more new possibilities for the early detection of PTVD and provide clues for more related gene mechanisms, therapeutic strategies and drug development, which is worthy of further study.

## Data Availability Statement

Due to ethical restrictions related to the consent given by subjects at the time of study commencement, our datasets are available from the corresponding author upon reasonable request after permission of the Institutional Review Board of State Key Laboratory of Cardiovascular Disease, Fuwai Hospital, National Center for Cardiovascular Diseases.

## Ethics Statement

The studies involving human participants were reviewed and approved by the Ethics Committee of Fuwai Hospital. The patients/participants provided their written informed consent to participate in this study.

## Author Contributions

XZ, JY, LS, RH, and RG contributed to the conception or design of the work. XZ drafted the manuscript. XT, JX, LJ, RL, YZ, DW, KS, BX, and WZ contributed to the data acquisition. KH, XT, JX, and JL contributed to the analysis, or interpretation of data of the work. JY and LS critically revised the manuscript. All authors gave final approval and agree to be accountable for all aspects of work ensuring integrity and accuracy, contributed to the article, and approved the submitted version.

## Funding

This work was supported by the National Key Research and Development Program of China (Grant Nos. 2016YFC1301300 and 2016YFC1301301), Young and middle-aged talents in the XPCC Science and Technology Project (2020CB012), Beijing Natural Science Foundation (7181008), and CAMS Innovation Fund for Medical Sciences (CIFMS) (2020-I2M-C&T-B-052).

## Conflict of Interest

The authors declare that the research was conducted in the absence of any commercial or financial relationships that could be construed as a potential conflict of interest.

## Publisher's Note

All claims expressed in this article are solely those of the authors and do not necessarily represent those of their affiliated organizations, or those of the publisher, the editors and the reviewers. Any product that may be evaluated in this article, or claim that may be made by its manufacturer, is not guaranteed or endorsed by the publisher.
